# *In silico* characterization of tandem repeats in *Trichophyton rubrum* and related dermatophytes provides new insights into their role in pathogenesis

**DOI:** 10.1093/database/bax035

**Published:** 2017-06-11

**Authors:** Matheus Eloy Franco, Tamires Aparecida Bitencourt, Mozart Marins, Ana Lúcia Fachin

**Affiliations:** 1 Unidade de Biotecnologia, Universidade de Ribeirão Preto, Av: Costabile Romano 2201, 14096-900, Ribeirao Preto SP, Brazil; 2 Federal Institute of Education, Science and Technology of South of Minas Gerais - IFSULDEMINAS, 37750-000, Brazil; 3 Departamento de Genetica, 049-900, FMRP-USP, SP, Brazil; 4 Curso de Medicina, Universidade de Ribeirão Preto, SP, Brazil

## Abstract

*Trichophyton rubrum* is the most common etiological agent of dermatophytoses worldwide, which is able to degrade keratinized tissues. The sequencing of the genome of different dermatophyte species has provided a large amount of data, including tandem repeats that may play a role in genetic variability and in the pathogenesis of these fungi. Tandem repeats are adjacent DNA sequences of 2–200 nucleotides in length, which exert regulatory and adaptive functions. These repetitive DNA sequences are found in different classes of fungal proteins, especially those involved in cell adhesion, a determinant factor for the establishment of fungal infection. The objective of this study was to develop a Dermatophyte Tandem Repeat Database (DTRDB) for the storage and identification of tandem repeats in *T. rubrum* and six other dermatophyte species. The current version of the database contains 35 577 tandem repeats detected in 16 173 coding sequences. The repeats can be searched using entry parameters such as repeat unit length (nt—nucleotide), repeat number, variability score, and repeat sequence motif. These data were used to study the relative frequency and distribution of repeats in the sequences, as well as their possible functions in dermatophytes. A search of the database revealed that these repeats occur in 22–33% of genes transcribed in dermatophytes where they could be involved in the success of adaptation to the host tissue and establishment of infection. The repeats were detected in transcripts that are mainly related to three biological processes: regulation, adhesion, and metabolism. The database developed enables users to identify and analyse tandem repeat regions in target genes related to pathogenicity and fungal–host interactions in dermatophytes and may contribute to the discovery of new targets for the development of antifungal agents.

**Database URL:** http://comp.mch.ifsuldeminas.edu.br/dtrdb/

## Introduction

Dermatophytes are a group of filamentous fungi that can invade and colonize keratinized tissues in humans and animals. Infections caused by these fungi are the most common in the world (1). Dermatophytes are specialized in infecting keratinized tissues such as nails, skin and hair and can be classified according to their preferred habitat as geophilic, zoophilic and anthropophilic (2). *Trichophyton rubrum* is an anthropophilic dermatophyte that is responsible for ∼70% of dermatophytoses in humans (3). An aggravating factor of infection with this dermatophyte is the fact that *T. rubrum* can cause invasive infections in immunocompromised patients, which can become deep and generalized infections (4). Because of their clinical importance, the genomes of *T. rubrum* and of six other species have been sequenced and are available at http://www.broadinstitute.org/annotation/genome/dermatophyte_comparative (5), recently upgraded in ENSEMBL FUNGI: http://fungi.ensembl.org. These data are important to increase our knowledge about key aspects of the virulence of dermatophytes, their ability to colonize specific niches, and host interactions. The availability of the genomes of these dermatophytes opens the possibility for different types of analysis, including the search for tandem repeat regions which are associated with virulence and environmental adaptation in some organisms (6).

Tandem repeats are hypervariable, sequentially repeated sequences that can be classified into microsatellites (1–9 bp) or minisatellites (≥10 bp) according to the length of the repeat unit (7). Tandem repeats play an important role in the regulation of gene expression and phenotypic variation and have been associated with pathogenicity in different microorganisms, particularly yeasts such as *Candida albicans* (6). In *Aspergillus fumigatus*, Levdansky et al. (8) showed that genes with tandem repeats play a key role in the pathogen–host interaction. The role of these repeats in dermatophyte fungi is still not well understood. However, it is believed that tandem repeats increase cell–cell aggregation, especially when they are found in regions that encode cell surface proteins such as adhesins. Minisatellites (>9 bp) present in these proteins can trigger recombination events and the formation of new adhesins, providing the fungus with a rich repertoire of properties, conferring phenotypic plasticity and permitting rapid adaptation to stressful environments (9). For example, in *Saccharomyces cerevisiae*, variations in repeat number were positively associated with the ability to increase cell adhesion (10). Richard and Dujon (11), studying minisatellite repeats, reported that 50–60% of the genes encoding cell wall and cell adhesion proteins in fungi contained this type of tandem repeat.

It should be noted that, because of their conservation in evolution, tandem repeats are not found in all genes, but rather tend to be present in genes that respond to changes in environmental conditions. Consequently, some of these tandem repeats can serve as a mechanism of adaptation to the environment by mediating phenotypic alterations and favoring pathogen–host interactions (7).

In dermatophytes, adhesins are the determinants of infection of the host cell and are therefore key factors for the virulence of these fungi (12). During the early stage of infection with dermatophytes, the conidia must overcome the innate defense mechanisms of the host and adhere to the epidermis, followed by germination of the arthroconidia and hyphal penetration of the stratum corneum. During the adhesion of arthroconidia to the surface of the stratum corneum, long fibrillar structures are formed, which seem to anchor and connect the arthroconidium to the tissue surface, preventing their removal from the host tissue (13).

Recently, microarray gene expression data of *T. rubrum* grown in culture medium with keratin have shown strong induction of a gene that encodes a hypothetical protein. *In silico* analysis of this sequence revealed an adhesin-like protein rich in tandem repeat sequences of glycine, glutamine and proline, which is characterized by the presence of mucin, flocculin and collagen domains. The similarity of the sequence of this protein with other cell surface proteins of pathogenic fungi such as *Aspergillus fumigatus* and *Metarhizium anisopliae*, which are potentially related to virulence, adhesion and germination, support the role of this putative adhesin in pathogen–host interactions. These data were further evaluated by gene expression analysis using quantitative PCR during the interaction of *T. rubrum* conidia with human keratinocytes. The results showed expressive induction of the gene encoding the putative adhesion at 6 and 24 h of fungal infection, suggesting its importance for virulence-related processes and fungus–host interactions (14).

Within this context, the objective of this study was to develop a Dermatophyte Tandem Repeat Database (DTRDB) and a pipeline for automation of the processes of identification and storage of these repeats using different technologies. This database was used to identify and analyse tandem repeat regions in target coding genes related to pathogenicity and parasite–host interactions in dermatophyte species, particularly *T. rubrum*.

## Materials and methods

### Construction of the database

The MySQL relational database management system was used for storage of the data. A front-end web interface was developed using web technologies such as HTML, CSS, JQuery and ASP.NET Web Forms (C# language) for communication with the database. The database was constructed using a 3-tier architecture, including the user interface, the code and the database. In addition to the tables responsible for storing the data, the database possesses SQL queries for manipulation of the data in stored procedures. The Entity Relationship Diagram is available as supplementary data (Supplementary Figure S1). DTRDB runs on a Windows Server 2012 operation system with the Microsoft IIS web server. The tools used for identification of tandem repeats in the pipeline run on an Ubuntu Linux server.

### Identification of repeats

The analysis was limited to tandem repeat arrangements in coding sequences. The Tandem Repeat Finder algorithm was used for the identification of intragenic repeats using sequences of transcribed genes present in public databases (15). The following parameters defined based on the studies of Legendre et al. (16) and Vinces et al. (17) were used: matching weight 2, mismatching penalty 5, indel penalty 5, match probability 0.8, indel probability 0.1, score ≥40, and maximum period 500. These parameters can be used to identify perfect and degenerate repeats. For analysis of repeat variability, a variability score was calculated for each repeat using the SERV algorithm (16). The repeats were divided into variability groups in which repeats with a score of 1 or higher (VARScore ≥ 1) are classified as highly mutable and repeats with a score between 0 and 1 as variable (18).

### Conservation of repeats

Conservation of the repeats between species was analysed by local alignment with the Blast tool using an e-value of 1e^−05^ (19). Repeats showing identity to at least one species were defined as conserved. The percentage of conservation was calculated by dividing the number of identity repeats by the total number of repeats in the organism.

### Sequences of transcribed genes

The fungal transcriptome of *Trichophyton rubrum* CBS 118892, *Trichophyton tonsurans* CBS 112818, *Trichophyton equinum* CBS 127.97, *Microsporum gypseum* CBS 118893, *Microsporum canis* CBS 113480, *Arthroderma benhamiae* CBS 112371, and *Trichophyton verrucosum* HKI 0517 analysed in this study were obtained from the Broad Institute internet site at http://www.broadinstitute.org/annotation/genome/dermatophyte_comparative in May 2014. These data are also available in public databases such as NCBI.

### Functional annotation

Functional annotations were generated for all transcripts of *T. rubrum* with variable tandem repeats using the Blast2Go tool (20) and stringent parameters (e-value of 1e^−05^). In addition, fungal adhesins were predicted using the FaaPred tool (12), with a threshold ≥0.5.

## Results and discussion

**Figure 1.**
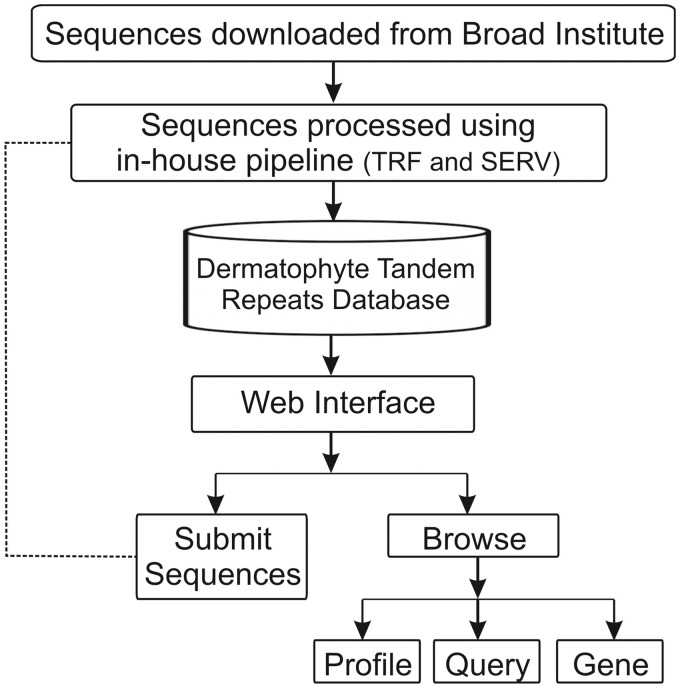
Schematic representation of the architecture of the Dermatophyte Tandem Repeat Database.

Using a web browser, the DTRDB database provides interactive access not only to the stored data, but also to a pipeline that automates the identification and storage of tandem repeats in submitted sequences available through an intranet (Figure 1). The database currently contains 35 577 tandem repeats identified in 16 173 sequences of coding genes of seven dermatophyte species. A web-based user interface divided into two main modules was developed: ‘Submit Sequences’ (intranet) and ‘Browse’ (open).

**Figure 2.**
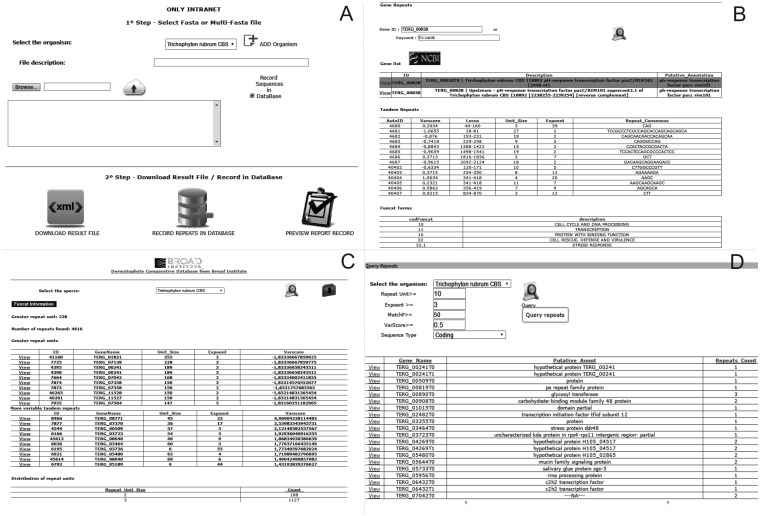
Screens of the web pipeline. (**A**) Submission form of the fasta file for the identification and storage of repetitive sequences. (**B**) Query of repeats and functional information of the dermatophyte *Trichophyton rubrum*. (**C**) Profile of repeats existing in a certain organism. (**D**) Query of repeats in an organism using filters.

The ‘Submit Sequences’ module enables to send sequences through the intranet for the identification and storage of tandem repeats (Figure 2A). The ‘Browse’ module provides three types of queries for the stored repeats: (i) ‘Profile Repeats’ enables visualization of the profile of stored tandem repeats by selecting a species. This profile contains information such as the number of repeats identified, genes with the most variable repeats and distribution of repeats per unit, and enables users to download the dataset of the stored data (Figure 2B). (ii) ‘Query Repeats’ permits to search genes containing repeats that meet entry parameters such as repeat unit, exponent (repeat unit copy number) and variability score. Once a gene has been selected, the repeats it contains are shown. A repeat can then be selected and it is verified whether this motif is found in any other gene stored in the database. Additionally, it is possible to access information of the selected gene through integration with the NCBI website (Figure 2C). (iii) ‘Search Gene Repeats’ enables to search repeats based on the gene identifier (Broad Institute pattern) or keyword present in its annotation (Figure 2D). In the case of *T. rubrum*, the stored functional categories according to the Gene Ontology (21), PFAM (22) and MIPS PEDANT Funcat (23) terms are also shown.

### Pipeline

The DTRDB allows to perform the following basic tasks: (i) identification of tandem repeats using a fasta file submitted via the web interface (intranet); (ii) storage of the repeats in a relational database; (iii) search of repeat patterns using filters such as unit size, length, and conservation; (iv) visualization of the repeat profile in a certain stored organism, and (v) search of functional information about genes of the dermatophyte *T. rubrum*. The pipeline (Figure 2) is available (the submission of files is only possible via an intranet) at http://comp.mch.ifsuldeminas.edu.br/dtrdb.

### Profile of tandem repeats in dermatophytes

The pipeline developed enabled us to identify, store and query tandem repeats in *T. rubrum* and related dermatophytes (*Trichophyton tonsurans*, *Trichophyton equinum*, *Microsporum gypseum*, *Microsporum canis*, *Arthroderma benhamiae*, and *Trichophyton verrucosum*) obtained from the Broad Institute internet site (2014).

As can be seen in Table 1, the number of repeats identified ranged from 3724 in *M. canis* to 6720 in *A. benhamiae*. No correlation was observed between the size or number of sequences and the number of tandem repeats. *T. rubrum* exhibited 4616 repeats in 10 416 transcribed genes (13.54 Mb), while 6720 repeats were identified in 7980 transcribed gene sequences (11.83 Mb) of *A. benhamiae*. Similar results have been reported by Mayer; Leese and Tollrian (24). A total of 4616 tandem repeats were identified in *T. rubrum* genome*.* However, the genome assembly is still incomplete and may suffer alterations. Furthermore, it should be taken into consideration that the quantifications and percentages presented are not accurate*.* The DTRDB database showed that these repeat are distributed across 2348 sequences of a total of 10 418 transcribed genes, corresponding to a repeat density of 22.53% in the sequences of transcribed genes. Of these 4616 repeats, 4191 were identified in 2075 hypothetical genes, while the remaining 425 repeats were identified in 273 previously annotated sequences. Thus, the tandem repeats were predominantly concentrated in hypothetical transcribed genes.

**Table 1.** Profile of tandem repeats in transcribed genes of dermatophytes

	*T. rubrum*	*T. tonsurans*	*T. equinum*	*M. gypseum*	*M. canis*	*T. verrucosum*	*A. benhamiae*
Size of transcribed genes (Mb^a^)	13.54	12.00	11.90	12.79	13.00	11.78	11.83
Number of transcribed genes^b^	10 418	8523	8679	8907	8915	8024	7980
Repeats	4616	4518	4634	4829	3724	6536	6720
Conservation^c^	19.5%	43.25%	42.25%	2.96%	2.44%	22.77%	22.02%
Largest repeat unit	228	405	309	378	296	220	233

a One million base pairs or megabase pair.

b Number of sequences of transcribed genes obtained from the Broad Institute site in October 2014.

c Percentage of repeat conservation in relation to the other species.

The pipeline enabled us to obtain the distribution of repeats according to repeat unit. Table 2 shows the number of repeat units that occurs at least 10 times in the coding gene sequence. The relative abundance in megabase was calculated by dividing the number of repeats by the size of the transcribed genes in megabase (Mb).

**Table 2.** Occurrence of tandem repeat units and relative abundance^a^

Unit	*T. rubrum*	*T. tonsurans*	*T. equinum*	*M. gypseum*	*M. canis*	*T. verrucosum*	*A. benhamiae*
2			11 (0.8)			74 (5.5)	91 (6.7)
3	471 (34.8)	536 (39.6)	527 (38.9)	392 (29)	259 (19.1)	848 (62.6)	902 (66.6)
4		17 (1.3)	23 (1.7)	19 (1.4)		65 (4.9)	67 (4.9)
5	14 (1.3)	10 (0.7)	20 (1.5)	25 (1.8)		70 (5.2)	59 (4.4)
6	459 (33.9)	498 (36.8)	513 (37.9)	554 (40.9)	271 (20.1)	684 (50.5)	710 (52.4)
7	14 (1.3)	27 (2)	29 (2.1)	34 (2.5)	12 (0.9)	68 (5.2)	67 (4.9)
8	24 (1.8)	42 (3.1)	45 (3.3)	39 (2.9)	23 (1.7)	136 (10.4)	122 (9.1)
9	578 (42.7)	561 (41.4)	561 (41.4)	584 (43.1)	420 (31.2)	728 (53.8)	713 (52.7)
10	54 (4)	62 (4.6)	77 (5.7)	71 (5.2)	50 (3.7)	139 (10.3)	127 (9.4)
11	101 (7.5)	108 (8)	115 (8.5)	122 (9.1)	124 (9.2)	221 (16.3)	241 (17.8)
12	927 (68.5)	874 (64.5)	881 (65.7)	919 (67.9)	757 (56)	1050 (77.5)	1087 (80.3)
13	153 (11.3)	119 (8.8)	128 (9.5)	147 (10.9)	130 (9.6)	192 (14.2)	203 (15)
14	129 (9.5)	119 (8.8)	127 (9.4)	136 (10.4)	109 (8.5)	186 (13.7)	189 (14)
15	497 (36.8)	460 (34)	464 (34.3)	501 (37.1)	423 (31.2)	576 (42.5)	584 (43.1)
16	92 (6.8)	74 (5.5)	75 (5.5)	71 (5.2)	67 (4.9)	116 (8.6)	132 (9.7)
17	44 (3.2)	46 (3.4)	54 (4)	56 (4.1)	53 (3.9)	89 (6.6)	89 (6.6)
18	359 (26.5)	299 (22.8)	316 (23.3)	352 (26)	298 (22.9)	395 (29.2)	402 (29.7)
19	33 (2.4)	27 (2)	31 (2.3)	26 (1.9)	23 (1.7)	54 (4)	75 (5.5)
20	30 (2.2)	25 (1.8)	28 (2.7)	43 (3.2)	21 (1.6)	57 (4.3)	61 (4.6)
21	211 (15.6)	190 (14.3)	199 (14.7)	191 (14.2)	191 (14.2)	220 (16.2)	212 (15.7)
22	20 (1.5)	29 (2.1)	26 (1.9)	40 (3)	22 (1.6)	43 (3.2)	45 (3.3)
23	23 (1.7)	18 (1.3)	16 (1.2)	24 (1.8)	20 (1.5)	37 (2.7)	36 (2.7)
24	121 (8.9)	105 (7.8)	99 (7.3)	120 (8.9)	129 (9.5)	144 (10.6)	148 (10.9)
25				12 (0.9)		21 (1.6)	18 (1.3)
26				11 (0.8)		20 (1.5)	19 (1.4)
27	53 (3.9)	46 (3.4)	45 (3.3)	66 (4.9)	45 (3.3)	64 (4.7)	55 (4.6)
28						11 (0.8)	20 (1.5)
29						11 (0.8)	
30	43 (3.2)	53 (3.9)	43 (3.2)	38 (2.9)	35 (2.6)	48 (3.5)	53 (3.9)
31							10 (0.7)
33	23 (1.7)	17 (1.3)	22 (1.6)	30 (2.2)	20 (1.5)	17 (1.3)	18 (1.3)
36	20 (1.5)	20 (1.5)	22 (1.6)	17 (1.3)	21 (1.6)	31 (2.3)	22 (1.6)
39				18 (1.3)	12 (0.9)	11 (0.8)	17 (1.3)
42	17 (1.3)	14 (1.3)	12 (0.9)	13 (1)		14 (1.3)	19 (1.4)
45	15 (1.2)	11 (0.8)		13 (1)			
48				14 (1.3)			
51			10 (0.7)				

a Relative abundance (in parentheses) is the total number of repeats per megabase of the sequence analysed. The table shows only tandem repeats where the repeat unit occurs at least 10 times (complete data is available in DTRDB).

**Figure 3.**
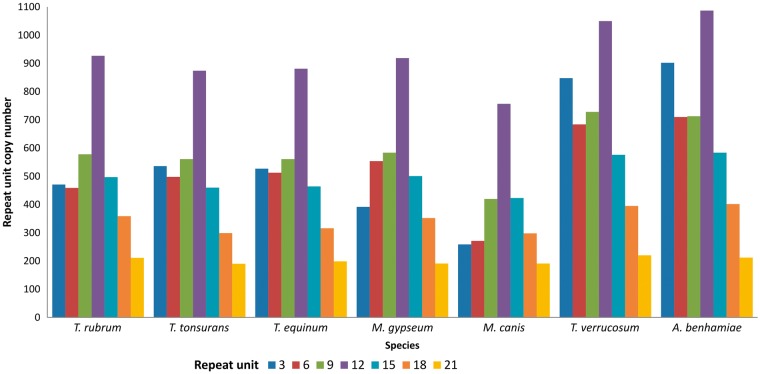
Distribution of tandem repeats according to repeat unit length (nt).

It can be observed that the largest number of tandem repeats in transcribed genes of dermatophytes are found in repeat units that are divisible by three. Consequently, the most prevalent repeats do not alter the reading frame, suggesting that they generate proteins with repetitive patterns (25). Indeed, Figure 3 shows that the repeats are mainly found in repeat units that are divisible by three, especially 3–21 bp, which account for ∼70% of all repeats in dermatophytes.

Different parameters have been used in studies investigating tandem repeats in different fungal species (26), but no studies are available for dermatophytes. Karaoglu and Meyer (27) conducted a survey of perfect short tandem repeats (1–6 bp per repeat unit) with a minimum length of 10 bp in the genome of nine fungal species using a Python-based algorithm specifically developed for their study. The authors identified 14 319 repeats in the genome of *Neurospora crassa* (38 Mb), with a relative abundance of 377 repeats per megabase. In contrast, another study identified 13 292 short repeats (1–6 bp per repeat unit) in the genome of *Neurospora crassa* using the Phobos tool developed by the authors; however, imperfect repeats were also considered (24).

The patterns of the most abundant tandem repeats in transcribed genes are similar in all dermatophytes. The CAG repeat is the most frequent in all dermatophyte species. The same was observed by Singh et al. (28) in the genome of *Puccinia triticina*. Huntley and Clark (29), who analysed the genome of 12 different organisms, found the CAG repeat to be the most prevalent in coding regions of the genome of *Drosophila*. Table 3 shows the most prevalent repeats (>20 occurrences) in transcribed genes of seven dermatophyte species.

**Table 3.** Most prevalent patterns of repeats

*T. rubrum*		
Repeat	Unit length	Number
CAG	3	125
GCA	3	51
CAA	3	35
CAGCAGCAA	9	28
AGC	3	25
*T. tonsurans*		
CAG	3	98
GCA	3	48
CAA	3	46
AGA	3	31
AGC	3	27
GAA	3	25
*T. equinum*		
CAG	3	96
CAA	3	46
GCA	3	43
GAA	3	31
AGA	3	28
AGC	3	26
*M. gypseum*		
CAG	3	81
GAA	3	38
GCA	3	34
AGA	3	29
CAA	3	22
*M. canis*		
CAG	3	71
GAA	3	25
*T. verrucosum*		
CAG	3	109
GAA	3	94
AGA	3	73
AAG	3	60
TCT	3	48
GCA	3	45
CAA	3	44
CTT	3	36
TTC	3	34
AGC	3	29
CTG	3	25
ACA	3	22
AG	2	22
CAGCAA	6	21
*A. benhamiae*		
CAG	3	123
GAA	3	105
AGA	3	73
AAG	3	56
TCT	3	47
CTT	3	46
CAA	3	42
GCA	3	42
TTC	3	33
CTG	3	30
AG	2	29
TC	2	26
CAGCAA	6	21

**Figure 4.**
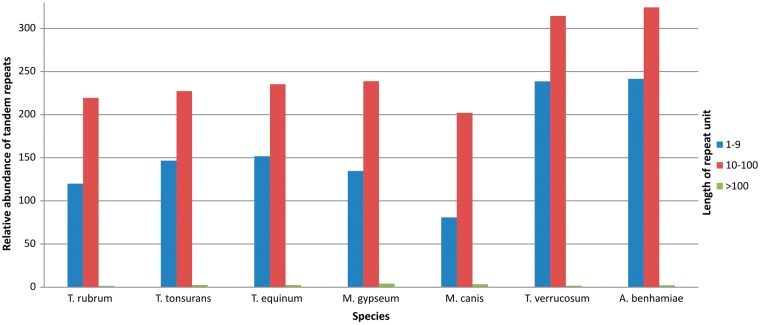
Relative abundance of grouped repeats.

Figure 4 shows the results grouped according to repeat unit lengths of 1–10 bp, 11–100 bp, and >100 bp. There was a predominance of minisatellites, especially considering repeats with <40 bp per unit. In addition, the number of repeats decreases with increasing unit length. This finding has also been reported by Gibbons and Rokas (30) who analysed tandem repeats in intragenic regions of 10 *Aspergillus* genomes.

### Variable number of tandem repeats in *Trichophyton rubrum*

**Figure 5.**
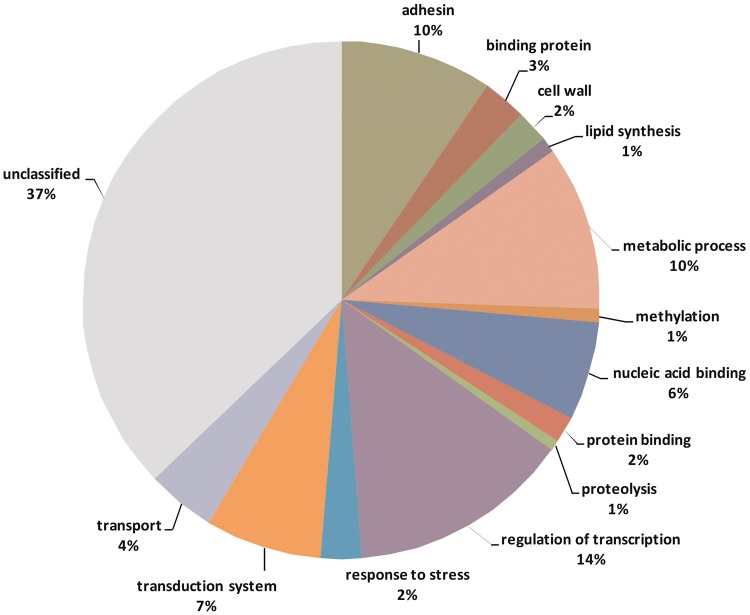
Functional categories of genes containing variable tandem repeats.

Genome studies on the sources of phenotypic variation have mainly focused on single nucleotide polymorphisms (SNPs) (31). In this study, we intended to identify and describe variable tandem repeats in *T. rubrum*. We hypothesized that these repeats can influence phenotypes by causing instability in important genes of this organism. Among 10 418 transcribed genes, 453 contain variable repeats (VARScore between 0 and 1) and 68 contain highly variable repeats (VARScore ≥ 1). Supplementary Table S1 (Supplemental Material) lists annotated (tentative) genes containing variable repeats and their respective functional categories. Table 4 shows the variation in tandem repeats between some genes of dermatophytes involved in different processes. The genes rich in variable repeats are related to different biological functions such as transcription factors, cell wall biosynthesis, and cell adhesion as shown in Figure 5.

**Table 4.** Variable repeats

*T. rubrum*			*T. tonsurans*	*T. equinum*	*M. gypseum*	*M. canis*	*T. verrucosum*	*A. benhamiae*
Gene name	Repeat	VARScore						
TERG_08771	45×23.4	4.97	45×5	45×9	No	No	45×4.5	No
TERG_00768	12×6.8	0.33	No	12×3.8	No	No	No	12×5.8
TERG_03736	6×54.7	1.77	6×39.7	6×44.7	6×48.7	No	6×28.7	No
TERG_05189	6×43.5	1.43	6×42.5	6×42.5	6×23.5	6×12.5	No	No
TERG_01042	3×27	1.11	3×15	3×15	No	No	3×20	3×12

### Tandem repeats in adhesins

Approximately 10% of all coding sequences of *T. rubrum* that contain tandem repeats were classified as adhesins by the FaaPred tool and these repeats are strongly related to the adhesion capacity of these proteins (10). Different known fungal adhesins are rich in variable tandem repeats and have been extensively studied in *Candida albicans*. In the *ALS* family of *C. albicans*, Hoyer et al. (32) found the number of copies of the tandem repeat in the central domain of each *ALS* gene to vary between isolates. Oh et al. (33) showed that adhesins with more repeat units have a greater adhesion capacity than those with fewer repeat units. In *Aspergillus fumigatus*, Levdansky et al. (34) demonstrated that genes containing tandem repeats play an important role in the pathogen–host interaction. The authors disrupted the *Afu3g08990* gene, which contains an 18-bp tandem repeat unit that repeats itself 32 times. Suppression of the protein previously characterized as hypothetical resulted in a phenotype with lower adhesion capacity.

## Conclusion

The results of the present study enabled the identification and categorization of different genes containing variable repetitive regions in *T. rubrum*. The genes rich in variable tandem repeats are related to different biological functions such as transcription factors, cell wall biosynthesis, and cell adhesion. The database for analysis of tandem repeats in dermatophytes allowed access to these repetitive patterns in coding regions of the genome of recently sequenced dermatophytes, permitting a better understanding of the nature and functional role of genes containing tandem repeats. The different tandem repeat patterns identified may reveal new molecular targets for the discovery of antifungal drugs and should increase our understanding of the role of these repetitive sequences in the pathogenicity of dermatophytes.

## Supplementary data

Supplementary data are available at *Database* Online.

## Supplementary Material

Supplementary Data
