# Supplementary material for: In silico characterization of tandem repeats in Trichophyton rubrum and related dermatophytes provides new insights into their role in pathogenesis
Source: Database (Oxford). 2017 Jun 11;2017:bax035. doi: 10.1093/database/bax035 (PMC5502367; doi:10.1093/database/bax035)
Supplement: Supplementary Data [file bax035_Supp.zip › Figure 1 Supplementary_Data- Entity Relationship Diagram.docx]

# SUPPLEMENTARY DATA


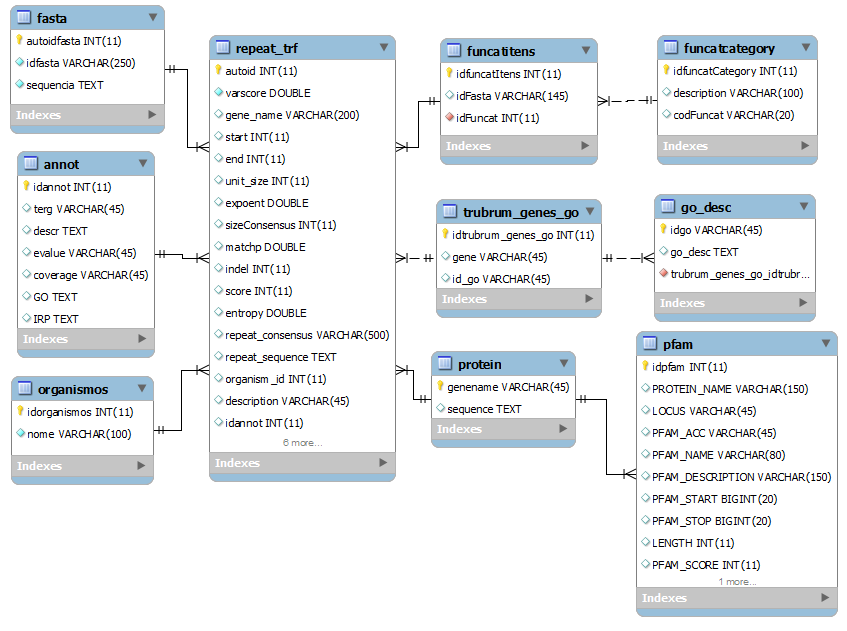


Supplementary Figure 1 - Entity Relationship Diagram

**Tables**

**repeat_trf:** Store the tandem repeats.

**organismos:** Store a description of strains.

**annot**: Store a putative annotation retrieved with Blast2GO

**fasta**: Store a name of fasta file sequences.

**funcatitens**: Stored funcat itens.

**funcatcategory**: Categories of funcat table.

**trubrum**_**genes**_**go**: Relation between GO terms and T. rubrum genes.

**go**_**desc**: Description of Gene Ontology itens.

**protein:** Aminoacids of gene translator.

**pfam:** Relation of pfam itens and T. rubrum proteins.
